# Supplementary figures and images for: PRECISE-DYAD: a prospective cohort study linking maternal and infant health trajectories in sub-Saharan Africa
Source: BMJ Open. 2026 Jul 17;16(7):e115586. doi: 10.1136/bmjopen-2025-115586 (PMC13384177; doi:10.1136/bmjopen-2025-115586)

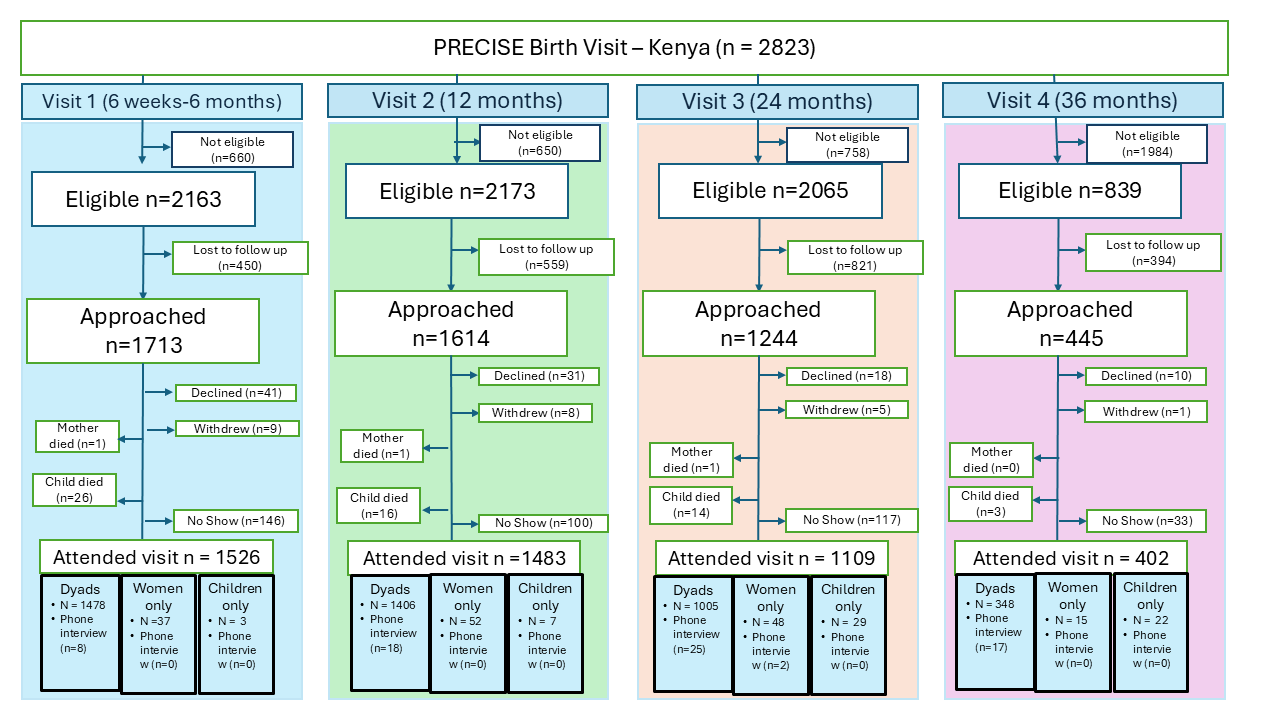

Supplement: online supplemental figure 1 [file bmjopen-16-7-s001.PNG]

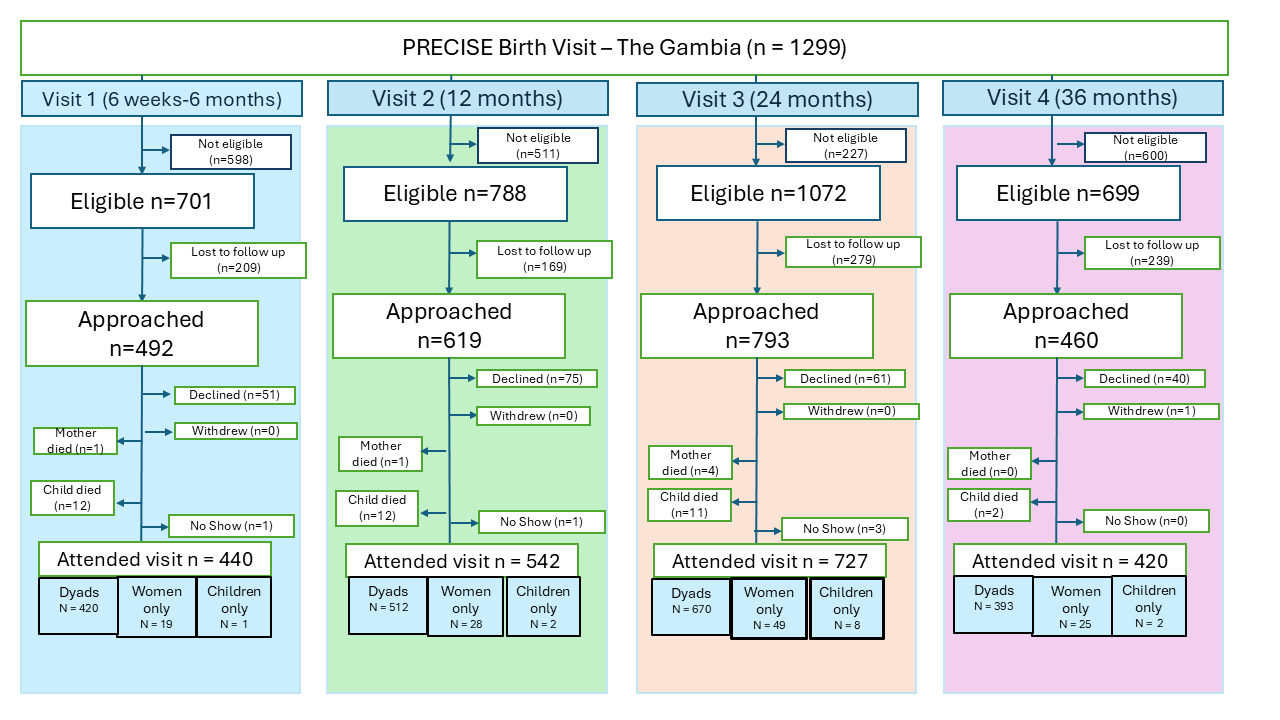

Supplement: online supplemental figure 2 [file bmjopen-16-7-s002.PNG]

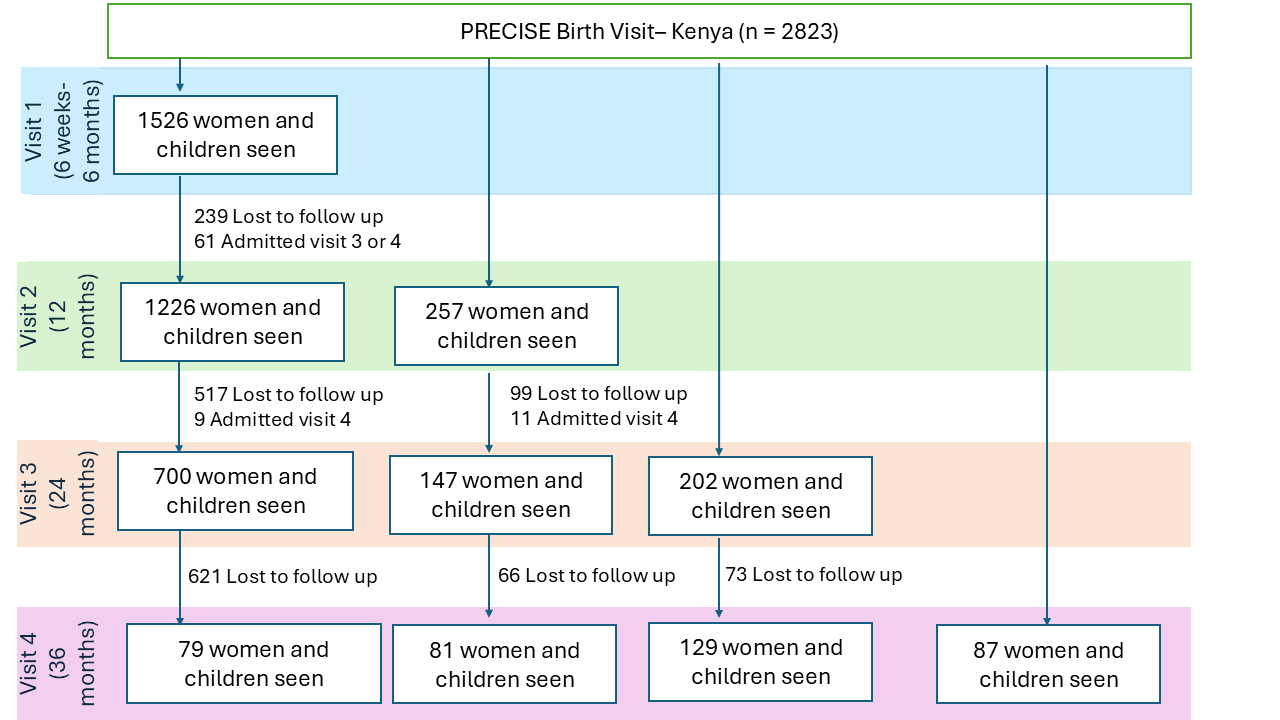

Supplement: online supplemental figure 3 [file bmjopen-16-7-s003.PNG]

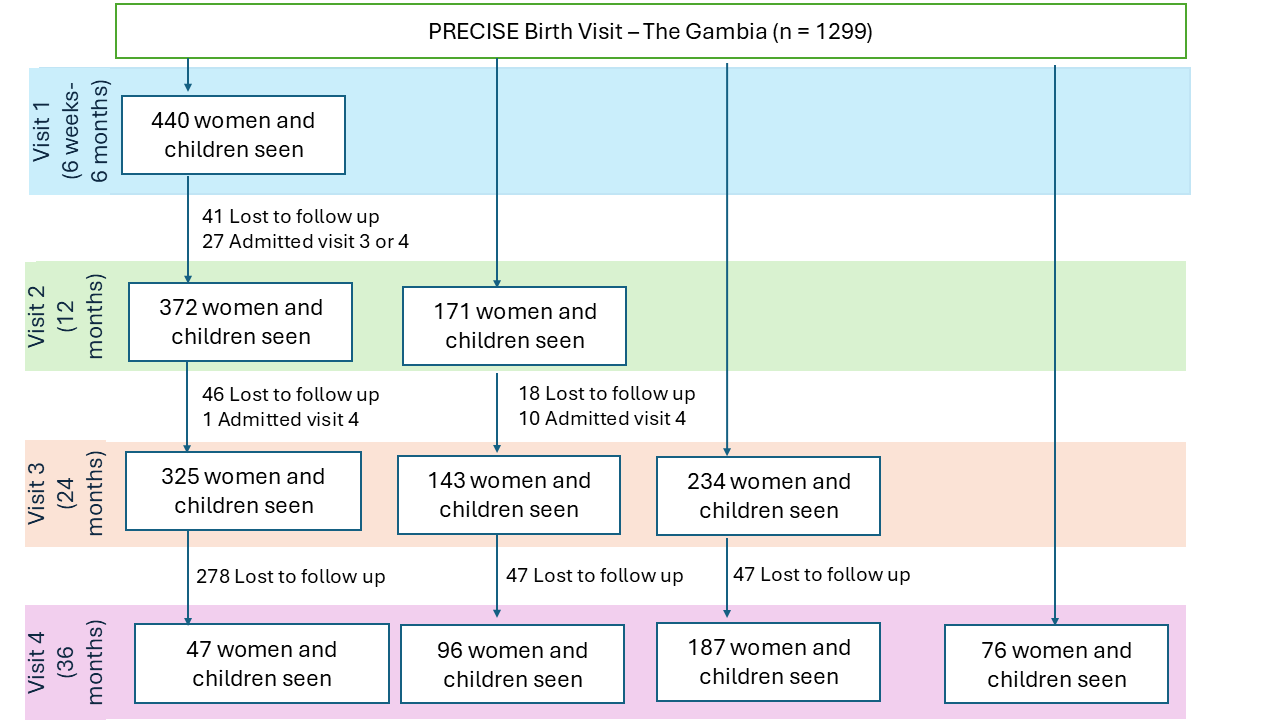

Supplement: online supplemental figure 4 [file bmjopen-16-7-s004.PNG]
