# Supplementary material for: PRECISE-DYAD: a prospective cohort study linking maternal and infant health trajectories in sub-Saharan Africa
Source: BMJ Open. 2026 Jul 17;16(7):e115586. doi: 10.1136/bmjopen-2025-115586 (PMC13384177; doi:10.1136/bmjopen-2025-115586)
Supplement: online supplemental table 1 [file bmjopen-16-7-s005.pdf]

Table S1 Pregnancy and birth outcomes of participants (from Kenya and The Gambia) at each PRECISE-DYAD visits.

	The Gambia				Kenya				All countries			
	Visit 1 (6 wk-6 months after birth)	Visit 2 (12 months after birth)	Visit 3 (24 months after birth)	Visit 4 (36 months after birth)	Visit 1 (6 wk-6 months after birth)	Visit 2 (12 months after birth)	Visit 3 (24 months after birth)	Visit 4 (36 months after birth)	Visit 1 (6 wk-6 months after birth)	Visit 2 (12 months after birth)	Visit 3 (24 months after birth)	Visit 4 (36 months after birth)
Number of women recruited in PRECISE-DYAD	**440**	**542**	**727**	**420**	**1526**	**1483**	**1109**	**402**	**1966**	**2025**	**1836**	**822**
Miscarriages (<20 weeks) N (%)	0 (0.0)	2 (0.4)	2 (0.3)	0 (0.0)	0 (0.0)	5 (0.3)	4 (0.4)	2 (0.5)	0 (0.0)	7 (0.3)	6 (0.3)	2 (0.2)
Maternal hypertension (HDP) N (%)	180 (40.9)	231 (42.6)	282 (38.8)	139 (33.1)	406 (26.6)	395 (26.6)	297 (26.8)	108 (26.9)	586 (29.8)	626 (30.9)	579 (31.5)	247 (30.1)
Maternal gestational hypertension	116 (26.4)	136 (25.1)	168 (23.1)	80 (19.0)	297 (19.5)	272 (18.3)	203 (18.3)	65 (16.2)	413 (21.0)	408 (20.1)	371 (20.2)	145 (17.6)
Maternal chronic hypertension	64 (14.5)	95 (17.5)	114 (15.7)	59 (14.0)	108 (7.1)	122 (8.2)	93 (8.4)	43 (10.7)	172 (8.7)	217 (10.7)	207 (11.3)	102 (12.4)
Maternal preeclampsia	75 (17.0)	92 (17.0)	110 (15.1)	55 (13.1)	133 (8.7)	134 (9.0)	95 (8.6)	39 (9.7)	208 (10.6)	226 (11.2)	205 (11.2)	94 (11.4)
Missing maternal hypertension outcome N (%)	21 (4.8)	2 (0.4)	1 (0.1)	0 (0.0)	49 (3.2)	2 (0.1)	1 (0.1)	1 (0.2)	70 (3.6)	4 (0.2)	2 (0.1)	1 (0.1)
Maternal death after birth	0	3	0	0	1	0	0	0	1	3	0	0
ICU admission N (%)	0 (0.0)	0 (0.0)	1 (0.1)	1 (0.2)	0 (0.0)	0 (0.0)	0 (0.0)	0 (0.0)	0 (0.0)	0 (0.0)	1 (0.1)	1 (0.1)
Number of children recruited in PRECISE-DYAD	**452)**	**559**	**746**	**427**	**1554**	**1515**	**1132**	**410)**	**2006**	**2074**	**1878**	**837**
GA at birth (weeks [IQR])	39.0 (37.1, 40.6)	39.0 (37.1,40.4)	39.1 (37.3, 40.6)	39.0 (37.3, 40.1)	39.1 (37.3, 40.6)	39.1 (37.2, 40.6)	39.0 (37.0, 40.6)	39.1 (37.6, 40.5)	39.1 (37.1, 40.6)	39.1 (37.1, 40.6)	39.0 (37.1, 40.6)	39.0 (37.6, 40.3)
Stillbirths N (%)	16 (3.5)	22 (3.9)	29 (3.9)	13 (3)	22 (1.4)	27 (1.8)	21 (1.9)	6 (1.5)	38 (1.9)	49 (2.4)	50 (2.7)	19 (2.3)
Infant death N (%)	6 (1.3)	8 (1.4)	17 (2.3)	9 (2.1)	24 (1.5)	32 (2.1)	31 (2.7)	11 (2.7)	30 (1.5)	40 (1.9)	48 (2.6)	20 (2.4)
Livebirths N (%)	436 (96.5)	535 (95.7)	714 (95.7)	413 (96.7)	1532 (98.6)	1482 (97.8)	1107 (97.8)	402 (98)	1968 (98.1)	2017 (97.3)	1821 (97)	815 (97.4)
Singleton N (%)	428 (94.7)	525 (93.9)	708 (94.9)	413 (96.7)	1498 (96.4)	1451 (95.8)	1086 (95.9)	394 (96.1)	1926 (96)	1976 (95.3)	1794 (95.5)	807 (96.4)
Twins N (%)	24 (5.3)	34 (6.1)	38 (5.1)	14 (3.3)	56 (3.6)	64 (4.2)	46 (4.1)	16 (3.9)	80 (4)	98 (4.7)	84 (4.5)	30 (3.6)
Small and vulnerable newborns (SVN) N (%)	191 (42.3)	229 (41)	279 (37.4)	147 (34.4)	565 (36.4)	546 (36)	423 (37.4)	123 (30)	756 (37.7)	775 (37.4)	702 (37.4)	270 (32.3)

Small for gestational age (SGA) N (%)	99 (21.9)	114 (20.4)	137 (18.4)	65 (15.2)	254 (16.3)	245 (16.2)	188 (16.6)	57 (13.9)	353 (17.6)	359 (17.3)	325 (17.3)	122 (14.6)
Missing SGA outcome	79 (17.5)	98 (17.5)	136 (18.2)	72 (16.9)	181 (11.6)	222 (14.7)	182 (16.1)	78 (19)	260 (13)	320 (15.4)	318 (16.9)	150 (17.9)
Preterm births N (%)	97 (21.5)	121 (21.6)	150 (20.1)	86 (20.1)	333 (21.4%)	325 (21.5)	255 (22.5)	70 (17.1)	430 (21.4%)	446 (21.5)	405 (21.6)	156 (18.6)
Missing preterm birth outcome	9 (2.0)	9 (1.6)	14 (1.9)	8 (1.9)	16 (1.0%)	20 (1.3)	10 (0.9)	3 (0.7)	25 (1.2)	29 (1.4)	24 (1.3)	11 (1.3)
Low birthweight N (%)	57 (12.6)	72 (12.9)	80 (10.7)	36 (8.4)	202 (13)	199 (13.1)	145 (12.8)	46 (11.2)	259 (12.9)	271 (13.1)	225 (12)	82 (9.8)
Missing low birth weight outcome	55 (12.2)	74 (13.2)	108 (14.5)	56 (13.1)	150 (9.7)	189 (12.5)	157 (13.9)	70 (17.1)	205 (10.2)	263 (12.7)	265 (14.1)	126 (15.1)
Low Apgar score N (%)	1 (0.2)	3 (0.5)	6 (0.8)	7 (1.6)	44 (2.8)	39 (2.6)	38 (3.4)	6 (1.5)	45 (2.2)	42 (2)	44 (2.3)	13 (1.6)
Missing Apgar score outcome	50 (11.1)	88 (15.7)	156 (20.9)	129 (30.2)	213 (13.7)	276 (18.2)	240 (21.2)	129 (31.5)	263 (13.1)	364 (17.6)	396 (21.1)	258 (30.8)
NICU admission N (%)	10 (2.2)	11 (2)	12 (1.6)	3 (0.7)	35 (2.3)	30 (2)	29 (2.6)	6 (1.5)	45 (2.2)	41 (2)	41 (2.2)	9 (1.1)
Missing NICU admission outcome	39 (8.6)	71 (12.7)	148 (19.8)	121 (28.3)	124 (8)	157 (10.4)	162 (14.3)	100 (24.4)	163 (8.1)	228 (11)	310 (16.5)	221 (26.4)

Table S2 Maternal demographic information and clinical characteristics of participants at each PRECISE-DYAD visits.

	The Gambia				Kenya				All countries			
	Visit 1 (6 wk-6 months after birth)	Visit 2 (12 months after birth)	Visit 3 (24 months after birth)	Visit 4 (36 months after birth)	Visit 1 (6 wk-6 months after birth)	Visit 2 (12 months after birth)	Visit 3 (24 months after birth)	Visit 4 (36 months after birth)	Visit 1 (6 wk-6 months after birth)	Visit 2 (12 months after birth)	Visit 3 (24 months after birth)	Visit 4 (36 months after birth)
**Number of participants (total)**	439	540	719	418	1523	1476	1079	380	1962	2016	1798	798
Number of participants (in person)	439	540	719	418	1515	1458	1053	363	1954	1998	1772	781
Number of phone interview	0	0	0	0	8	18	27	17	8	18	27	17
**Interval of time between birth and visit (months), median (IQR)**	5.5 (5.0, 5.8)	11.8 (11.1, 12.6)	24.0 (23.5, 24.6)	35.9 (35.4, 36.4)	3.6, 3.3 (3.0, 4.1)	11.4, 11.1 (11.0, 11.7)	23.1 (23.0, 23.6)	35.1 (35.0, 35.5)	3.5 (3.1, 5.0)	11.2 (11.0, 12.0)	23.4 (23.0, 24.2)	35.5 (35.1, 36.2)
**Maternal age, years median (IQR)**	28 (23, 32)	28 (23, 32)	29 (24, 33)	30 (25, 34)	27 (23, 32)	28 (24, 33)	30 (26, 34)	31 (27, 36)	27 (23, 32)	28 (24, 33)	29 (25, 34)	30 (26, 35)
**Age category, years** N (%)												
15-19	26 (5.9)	31 (5.7)	14 (1.9)	2 (0.5)	54 (3.5)	38 (2.6)	4 (0.4)	1 (0.3)	80 (4.1)	69 (3.4)	18 (1.0)	3 (0.4)
20-24	113 (25.7)	131 (24.2)	167 (23.2)	84 (20.1)	425 (27.9)	358 (24.3)	177 (16.4)	43 (11.3)	538 (27.4)	489 (24.3)	344 (19.1)	127 (15.9)
25-29	130 (29.6)	155 (28.7)	203 (28.2)	118 (28.2)	459 (30.1)	446 (30.2)	345 (31.9)	126 (33.2)	589 (30.0)	601 (29.8)	548 (30.5)	244 (30.6)
30-34	92 (21.0)	120 (22.2)	182 (25.3)	115 (27.5)	342 (22.5)	355 (24.1)	293 (27.1)	91 (23.9)	434 (22.1)	475 (23.6)	475 (26.4)	206 (25.8)
35-39	50 (11.4)	69 (12.8)	100 (13.9)	61 (14.6)	191 (12.5)	221 (15.0)	197 (18.2)	84 (22.1)	241 (12.3)	290 (14.4)	297 (16.5)	145 (18.2)
40-44	25 (5.7)	30 (5.5)	46 (6.4)	34 (8.1)	42 (2.8)	47 (3.2)	52 (4.8)	32 (8.4)	67 (3.4)	77 (3.8)	98 (5.4)	66 (8.3)
45-49	2 (0.5)	1 (0.2)	3 (0.4)	2 (0.5)	6 (0.4)	10 (0.7)	10 (0.9)	3 (0.8)	8 (0.4)	11 (0.5)	13 (0.7)	5 (0.6)
50+	0 (0.0)	1 (0.2)	1 (0.1)	1 (0.2)	1 (0.1)	0 (0.0)	1 (0.1)	0 (0.0)	1 (0.1)	1 (0.0)	2 (0.1)	1 (0.1)
missing	1 (0.2)	2 (0.4)	3 (0.4)	1 (0.2)	3 (0.2)	1 (0.1)	0 (0.0)	0 (0.0)	4 (0.2)	3 (0.1)	3 (0.2)	1 (0.1)
**Marital status** N (%)												
Married/ Co-habiting	434 (98.9)	532 (98.5)	701 (97.5)	408 (97.6)	1403 (92.2)	1360 (92.1)	1001 (92.8)	360 (94.7)	1837 (93.6)	1892 (93.8)	1702 (94.7)	768 (96.2)
Never married (or single)	5 (1.1)	8 (1.5)	15 (2.1)	8 (1.9)	94 (6.2)	94 (6.4)	64 (5.9)	16 (4.2)	99 (5.0)	102 (5.1)	79 (4.4)	24 (3.0)
Separated/Divorced/Widowed	0 (0.0)	0 (0.0)	3 (0.4)	2 (0.5)	24 (1.6)	21 (1.4)	14 (1.3)	4 (1.1)	24 (1.2)	21 (1.0)	17 (0.9)	6 (0.8)
missing	0 (0.0)	0 (0.0)	0 (0.0)	0 (0.0)	2 (0.1)	1 (0.1)	0 (0.0)	0 (0.0)	2 (0.1)	1 (0.0)	0 (0.0)	0 (0.0)

**Education** N (%)												
Higher	21 (4.8)	22 (4.1)	28 (3.9)	15 (3.6)	209 (13.7)	207 (14.0)	133 (12.3)	38 (10.0)	230 (11.7)	229 (11.4)	161 (9.0)	53 (6.6)
None	272 (62.0)	348 (64.4)	466 (64.8)	269 (64.4)	118 (7.8)	110 (7.5)	76 (7.0)	25 (6.6)	390 (19.9)	458 (22.7)	542 (30.1)	294 (36.8)
Primary	68 (15.5)	84 (15.6)	116 (16.1)	76 (18.2)	781 (51.3)	742 (50.3)	583 (54.0)	216 (56.8)	849 (43.3)	826 (41.0)	699 (38.8)	292 (36.6)
Secondary	78 (17.8)	86 (15.9)	109 (15.2)	58 (13.9)	413 (27.1)	416 (28.2)	287 (26.6)	101 (26.6)	491 (25.0)	502 (24.9)	396 (22.0)	159 (19.9)
Missing	0 (0.0)	0 (0.0)	0 (0.0)	0 (0.0)	2 (0.1)	1 (0.1)	0 (0.0)	0 (0.0)	2 (0.1)	1 (<0.1)	0 (0.0)	0 (0.0)
**Occupation** N (%)												
Business	0 (0.0)	0 (0.0)	0 (0.0)	0 (0.0)	141 (9.3)	140 (9.5)	94 (8.7)	41 (10.8)	141 (7.2)	140 (6.9)	94 (5.2)	41 (5.1)
Construction	0 (0.0)	0 (0.0)	0 (0.0)	0 (0.0)	0 (0.0)	0 (0.0)	0 (0.0)	0 (0.0)	0 (0.0)	0 (0.0)	0 (0.0)	0 (0.0)
Factory	0 (0.0)	0 (0.0)	0 (0.0)	0 (0.0)	29 (1.9)	28 (1.9)	30 (2.8)	15 (3.9)	29 (1.5)	28 (1.4)	30 (1.7)	15 (1.9)
Housewife	384 (87.5)	478 (88.5)	643 (89.4)	373 (89.2)	803 (52.7)	772 (52.3)	567 (52.5)	204 (53.7)	1187 (60.5)	1250 (62.0)	1210 (67.3)	577 (72.3)
Informal - Employment	0 (0.0)	0 (0.0)	0 (0.0)	0 (0.0)	200 (13.1)	206 (14.0)	144 (13.3)	47 (12.4)	200 (10.2)	206 (10.2)	144 (8.0)	47 (5.9)
Large-scale agriculture	4 (0.9)	4 (0.7)	5 (0.7)	4 (1.0)	0 (0.0)	0 (0.0)	0 (0.0)	0 (0.0)	4 (0.2)	4 (0.2)	5 (0.3)	4 (0.5)
Market trader	17 (3.9)	17 (3.1)	22 (3.1)	12 (2.9)	177 (11.6)	164 (11.1)	124 (11.5)	39 (10.3)	194 (9.9)	181 (9.0)	146 (8.1)	51 (6.4)
Other (specify)	20 (4.6)	25 (4.6)	33 (4.6)	25 (6.0)	18 (1.2)	15 (1.0)	10 (0.9)	3 (0.8)	38 (1.9)	40 (2.0)	43 (2.4)	28 (3.5)
Professional	8 (1.8)	10 (1.9)	10 (1.4)	3 (0.7)	125 (8.2)	118 (8.0)	86 (8.0)	27 (7.1)	133 (6.8)	128 (6.3)	96 (5.3)	30 (3.8)
Student	6 (1.4)	6 (1.1)	6 (0.8)	1 (0.2)	28 (1.8)	31 (2.1)	23 (2.1)	4 (1.1)	34 (1.7)	37 (1.8)	29 (1.6)	5 (0.6)
missing	0 (0.0)	0 (0.0)	0 (0.0)	0 (0.0)	2 (0.1)	2 (0.1)	1 (0.1)	0 (0.0)	2 (0.1)	2 (0.1)	1 (0.1)	0 (0.0)
**Mother started employment / returned to school since giving birth** N (%)												
Yes	110 (25.1)	132 (24.4)	302 (42.0)	177 (42.3)	468 (30.7)	730 (49.5)	631 (58.4)	230 (60.5)	578 (29.5)	863 (42.8)	933 (51.9)	407 (51.0)
**Household composition** N (%)												
Total Number of people in the household	13.0 (9.0, 18.0)	13.0 (9.0, 19.0)	13.0 (10.0, 19.0)	13.0 (9.0, 18.0)	4.0 (3.0, 6.0)	4.0 (3.0, 6.0)	4.0 (3.0, 7.0)	5.0 (3.0, 6.0)	5.0 (3.0, 9.0)	5.0 (3.0, 9.0)	7.0 (4.0, 12.0)	8.0 (5.0, 13.0)
Total number of people over 18	6 (4, 10)	6 (5, 10)	6 (4, 9)	6 (4, 10)	2 (2, 3)	2 (2, 3)	2 (2, 3)	2 (2, 3)	2.0 (2.0, 5.0)	3.0 (2.0, 5.0)	3.0 (2.0, 6.0)	4.0 (2.0, 6.0)

Total number of people under 18	6.0 (4.0, 10.0)	6.0 (4.0, 10.0)	6.0 (4.0, 10.0)	6.0 (4.0, 10.0)	2.0 (1.0, 3.0)	2.0 (1.0, 3.0)	2.0 (1.0, 3.0)	2.0 (1.0, 3.0)	2.0 (1.0, 4.0)	3.0 (1.0, 5.0)	3.0 (2.0, 6.0)	4.0 (2.0, 7.0)
Father living with the child	341 (77.7)	433 (80.2)	552 (76.8)	330 (78.9)	1279 (84.0)	1245 (84.3)	852 (78.9)	305 (80.3)	1620 (82.6)	1678 (83.2)	1404 (78.0)	635 (79.6)
Missing	12 (2.7)	13 (2.4)	23 (3.2)	8 (1.9)	24 (1.6)	23 (1.6)	43 (4.0)	12 (3.2)	36 (1.8)	36 (1.8)	66 (3.7)	20 (2.5)
Number of mother who were pregnant at the visit	6 (1.4)	15 (2.8)	149 (20.6)	55 (13.2)	8 (0.5)	38 (2.6)	83 (7.7)	25 (6.6)	14 (0.7)	53 (2.6)	231 (12.8)	80 (10.0)
**Maternal BMI, median (IQR)**	21.5 [19.2-25.3]	21.3 [18.7-25.0]	22.0 [19.2-25.2]	21.8 [19.4-24.5]	23.6 [20.9-27.5]	23.5 [20.3-27.9]	23.6 [20.4-28.2]	24.6 [21.0-28.9]	23.3 [20.4-27.0]	22.9 [19.8-26.9]	22.9 [19.8-27.1]	22.9 [19.9-26.7]
**Maternal BMI Category** N (%)												
<18.5	80 (18.2)	118 (21.9)	121 (16.8)	75/418 (17.9)	111/1515 (7.3)	161/1458 (11.0)	113/1053 (10.7)	33/363 (9.1)	191/1954 (9.8)	279 (14.0)	234 (13.2)	108/781 (13.8)
18.5-24.9	237 (54.0)	287 (53.1)	400 (55.7)	252/418 (60.3)	814/1515 (53.7)	720/1458 (49.4)	493/1053 (46.8)	157/363 (43.3)	1051/1954 (53.8)	1007 (50.4)	893 (50.4)	409/781 (52.4)
25-29.9	77 (17.5)	88 (16.3)	131 (18.2)	63/418 (15.1)	367/1515 (24.2)	327/1458 (22.4)	255/1053 (24.2)	97/363 (26.7)	444/1954 (22.7)	415 (20.8)	386 (21.8)	160/781 (20.5)
30	42 (9.6)	44 (8.1)	55 (7.6)	23/418 (5.5)	216/1515 (14.3)	244/1458 (16.7)	188/1053 (17.9)	71/363 (19.6)	258/1954 (13.2)	288 (14.4)	243 (13.7)	94/781 (12.0)
Missing	3 (0.7)	3 (0.6)	12 (1.7)	5/418 (1.2)	7/1515 (0.5)	6/1458 (0.4)	4/1053 (0.4)	5/363 (1.4)	10/1954 (0.5)	9 (0.5)	16 (0.9)	10/781 (1.3)
**MUAC- median (IQR)**	27.3 [24.8-30.3]	27.0 [24.4-30.3]	27.0 [24.6-30.0]	27.1 [24.7-30.0]	27.1 [24.6-30.2]	27.1 [24.5-30.5]	27.2 [24.5-31.0]	27.6 [24.6-31.5]	27.1 [24.6-30.2]	27.1 [24.5-30.5]	27.1 [24.5-30.4]	27.3 [24.7-30.5]
**MUAC Category** N (%)												
Underweight (<23.0cm)	47 (10.7)	61 (11.3)	83 (11.5)	33/418 (7.9)	157/1515 (10.4)	191/1458 (13.1)	127/1053 (12.1)	39/363 (10.7)	204/1954 (10.4)	252 (12.6)	210 (11.8)	72/781 (9.2)
Normal weight (23.0-31.9cm)	313 (71.3)	394 (73.0)	525 (73.0)	326/418 (78.0)	1120/1515 (73.9)	1003/1458 (68.8)	721/1053 (68.5)	240/363 (66.1)	1433/1954 (73.3)	1397 (69.9)	1246 (70.3)	566/781 (72.4)
Overweight/obese (≥32.0cm)	78 (17.8)	85 (15.7)	107 (14.9)	56/418 (13.4)	237/1515 (15.6)	262/1458 (18.0)	202/1053 (19.2)	81/363 (22.3)	315/1954 (16.1)	347 (17.4)	309 (17.4)	137/781 (17.5)
Missing	1 (0.2)	0 (0.0)	4 (0.6)	3/418 (0.7)	1/1515 (0.1)	2/1458 (0.1)	3/1053 (0.3)	3/363 (0.8)	2/1954 (0.1)	2 (0.1)	7 (0.4)	6/781 (0.8)
BP Category N (%)												
Normal	328 (74.7)	438 (81.1)	637 (88.6)	351 (84.0)	1057 (69.8)	1109 (76.1)	811 (77.0)	271 (74.7)	1385 (70.9)	1547 (77.4)	1448 (81.7)	622 (79.6)

Elevated	18 (4.1)	25 (4.6)	22 (3.1)	12 (2.9)	90 (5.9)	73 (5.0)	40 (3.8)	19 (5.2)	108 (5.5)	98 (4.9)	62 (3.5)	31 (4.0)
Stage 1 hypertension	69 (15.7)	63 (11.7)	47 (6.5)	41 (9.8)	288 (19.0)	224 (15.4)	166 (15.8)	57 (15.7)	357 (18.3)	287 (14.4)	213 (12.0)	98 (12.5)
Stage 2 hypertension	24 (5.5)	13 (2.4)	10 (1.4)	14 (3.3)	79 (5.2)	51 (3.5)	34 (3.2)	14 (3.9)	103 (5.3)	64 (3.2)	44 (2.5)	28 (3.6)
missing	0 (0.0)	1 (0.2)	3 (0.4)	0 (0.0)	1 (0.1)	1 (0.1)	2 (0.2)	2 (0.6)	1 (0.1)	2 (0.1)	5 (0.3)	2 (0.3)
**Cardiology assessment**												
Pulse wave velocity	-	7.1 (6.5, 8.2)		-	-	7.1 (6.6, 7.9)		-	-	7.1 (6.5, 7.9)		-
Cardiac Output	-	4.2 (3.4, 4.9)		-	-	4.8 (4.0, 5.8)		-	-	4.7 (3.8, 5.6)		-
Systemic Vascular Resistance	-	1578.9 (1355.8, 2020.9)		-	-	1401.5 (1150.6, 1774.8)		-	-	1456.2 (1201.4, 1840.3)		-
**Nutrition Status** N (%)												
Meets minimum dietary diversity	325 (74.0)	439 (81.3)	610 (84.8)	360/418 (86.1)	792/1515 (52.3)	825/1458 (56.6)	607/1053 (57.6)	207/363 (57.0)	1117/1954 (57.2)	1264/1998	1217/1772 ()	567/781 (72.6)
missing	0	0	0	0	0	0	0	0	0	0	0	0
**Mental health** N (%)												
Number of participants who screened positive for WHODAS	0/439 (0.0)	0/3 (0)	1/370 (0.3)	1/288 (0.3)	21/1515 (1.4)	46/704 (6.5)	50/847 (5.9)	20/323 (6.2)	21/1954 (1.1)	46/707 (6.5)	51/1217 (4.2)	21/611 (3.4)
Number of participants who screened positive for anxiety	5/438 (1.1)	1/370 (0.3)	2/365 (0.5)	0/287 (0)	13/544 (2.4)	46/866 (5.3)	29/771 (3.8)	5/251 (2.0)	18/982 (1.8)	47/1236 (3.8)	31/1136 (2.7)	5/538 (0.9)
Number of participants who screened positive for depression	3/438 (0.7)	0/370 (0)	3/365 (0.8)	1/287 (0.3)	7/544 (1.3)	21/866 (2.4)	12/771 (1.6)	4/251 (1.6)	10/982 (1.0)	21/1236 (1.7)	15/1136 (1.3)	5/538 (0.9)
Number of participants who screened positive for post traumatic stress	0/3 (0)	2/365 (0.5)	1/22 (4.5)	1/16 (6.3)	2/ 29 (6.9)	24/473 (5.1)	13/121 (10.7)	3/31 (9.7)	2/32 (6.3)	26/838 (3.1)	14/143 (9.8)	4/47 (8.5)
Number of participants who had suicidal thoughts	5/438 (1.1)	2/370 (0.5)	4/365 (1.1)	2/287 (0.7)	14/544 (2.6)	60/866 (6.9)	46/771 (6.0)	16/251 (6.4)	19/982 (1.9)	62/1236 (5.0)	50/1136 (4.4)	18/538 (3.3)

Table S3 Children clinical profile by visit

	The Gambia				Kenya				All countries			
	**Visit 1 (6 wk-6 months after birth)**	**Visit 2 (12 months after birth)**	**Visit 3 (24 months after birth)**	**Visit 4 (36 months after birth)**	**Visit 1 (6 wk-6 months after birth)**	**Visit 2 (12 months after birth)**	**Visit 3 (24 months after birth)**	**Visit 4 (36 months after birth)**	**Visit 1 (6 wk-6 months after birth)**	**Visit 2 (12 months after birth)**	**Visit 3 (24 months after birth)**	**Visit 4 (36 months after birth)**
**Number of children**	433	529	692	397	1511	1453	1076	391	1944	1982	1768	788
Girls N (%)	212 (49.0)	264 (49.9)	348 (50.3)	197 (49.6)	751 (49.7)	710 (48.9)	522 (48.5)	170 (43.5)	963 (49.5)	974 (49.1)	870 (49.2)	367 (46.6)
Boys N (%)	219 (50.6)	263 (49.7)	344 (49.7)	200 (50.4)	757 (50.1)	737 (50.7)	548 (50.9)	218 (55.8)	976 (50.2)	1000 (50.5)	892 (50.5)	418 (53.0)
Missing	2 (0.5)	2 (0.4)	0 (0.0)	0 (0.0)	3 (0.2)	6 (0.4)	6 (0.6)	3 (0.8)	5 (0.3)	8 (0.4)	6 (0.3)	3 (0.6)
**GA at birth (weeks [IQR])**	39.1 (37.4, 40.6)	39.0 (37.3, 40.6)	39.1 (37.7, 40.6)	39.0 (37.5, 40.1)	39.1 (37.3, 40.6)	39.1 (37.6, 40.6)	39.1 (37.3, 40.6)	39.1 (37.9, 40.6)	39.1 (37.3, 40.6)	39.1 (37.4, 40.6)	39.1 (37.4, 40.6)	39.1 (37.6, 40.3)
**Age(months) - Median (IQR)**	5.5 [5.0-5.8]	11.8 [11.1-12.6]	24.0 [23.5-24.6]	35.9 [35.4-36.4]	3.3 [3.0-4.0]	11.1 [11.0-11.7]	23.1 [23.0-23.6]	35.1 [35.0-35.5]	3.5 [3.1-5.0]	11.2 [11.0-12.0]	23.4 [23.0-24.2]	35.4 [35.1-36.1]
**Child health** N (%)												
Hospital admission												
yes	9/433 (2.1)	10/529 (1.9)	23/692 (3.3)	8/397 (2.0)	37/1503 (2.5)	95/1435 (6.6)	94/1051 (8.9)	26/374 (7.0)	46/1936 (2.4)	105/1964 (5.3)	117/1743 (6.7)	34/771 (4.4)
missing	3/433 (0.7)	6/529 (1.1)	8/692 (1.2)	7/397 (1.8)	6/1503 (0.4)	7/1435 (0.5)	5/1051 (0.5)	0/374 (0.0)	9/1936 (0.5)	13/1964 (0.7)	13/1743 (0.7)	16/771 (2.1)
Hospital stay length- days- median (IQR)	4.5 [2.3-5.0]	5.0 [3.3-8.0]	3.0 [2.0-7.5]	8.5 [6.0-14.8]	9.0 [4.0-14.0]	5.0 [3.0-7.0]	4.0 [3.0-7.0]	3.0 [3.0-6.8]	6.0 [3.0-7.3]	5.0 [3.0-7.0]	4.0 [2.0-7.0]	4.5 [3.0-8.8]
Malaria test	14/433 (3.2)	32/529 (6.0)	77/692 (11.1)	38/397 (9.6)	192/1503 (12.8)	729/1435 (50.8)	709/1051 (67.5)	241/374 (64.4)	206/1936 (10.6)	761/1964 (38.7)	786/1743 (45.1)	279/771 (36.2)
Test result positive	0/433 (0.0)	0/529 (0.0)	0/692 (0.0)	2/397 (0.5)	12/1503 (0.8)	76/1435 (5.3)	102/1051 (9.7)	49/374 (13.1)	12/1936 (0.6)	76/1964 (3.9)	102/1743 (5.9)	51/771 (6.6)
Child has difficulty seeing	-	5/529 (0.9)	0/692 (0.0)	1/397 (0.3)	-	7/1435 (0.5)	4/1051 (0.4)	2/374 (0.5)	-	12/1964 (0.6)	4/1743 (0.2)	3/771 (0.4)
Child has difficulty hearing	-	4/529 (0.8)	0/692 (0.0)	0/397 (0.0)	-	12/1435 (0.8)	3/1051 (0.3)	0/374 (0.0)	-	16/1964 (0.8)	3/1743 (0.2)	0/771 (0.0)
Child cough when no fever or illness	-	94/529 (17.8)	86/692 (12.4)	31/397 (7.8)	-	353/1435 (24.6)	223/1051 (21.2)	93/374 (24.9)	-	447/1964 (22.8)	309/1743 (17.7)	124/771 (16.1)
Child has wheezing or whistling in the chest	-	22/529 (4.2)	26/692 (3.8)	9/397 (2.3)	-	134/1435 (9.3)	143/1051 (13.6)	45/374 (12.0)	-	156/1964 (7.9)	169/1743 (9.7)	54/771 (7.0)

**Blood Pressure** N (%)												
BP ≥90th percentile	-	183/529 (34.6)	515/692 (74.4)	223/397 (56.2)	-	194/1435 (13.5)	607/1051 (57.8)	246/374 (65.8)	-	377/1964 (19.2)	1122/1743 (64.4)	469/771 (60.8)
BP <10th percentile	-	18/529 (3.4)	0/692 (0.0)	1/397 (0.3)	-	228/1435 (15.9)	7/1051 (0.7)	1/374 (0.3)	-	246/1964 (12.5)	7/1743 (0.4)	2/771 (0.3)
**Child nutrition Status** N (%)												
children breastfed exclusively	244/433 (56.4)	-	-	-	1187/1511 (78.6)	-	-	-	1431/1944 (73.6)	-	-	-
never breastfed	4/433 (0.9)	-	-	-	9/1511 (0.6)	-	-	-	13/1944 (0.7)	-	-	-
Still breastfeeding (0)	-	69/529 (13.0)	23/692 (3.3)	2/397 (0.5)	-	34/1435 (2.4)	8/1051 (0.8)	0/374 (0.0)	-	103/1964 (5.2)	31/1743 (1.8)	2/771 (0.3)
stopped exclusive breastfeeding 0-3 month (1)	-	48/529 (9.1)	55/692 (7.9)	36/397 (9.1)	-	68/1435 (4.7)	52/1051 (4.9)	16/374 (4.3)	-	116/1964 (5.9)	107/1743 (6.1)	52/771 (6.7)
stopped exclusive breastfeeding 4-6 month (2)	-	282/529 (53.3)	392/692 (56.6)	215/397 (54.2)	-	1044/1435 (72.8)	732/1051 (69.6)	259/374 (69.3)	-	1326/1964 (67.5)	1124/1743 (64.5)	474/771 (61.5)
stopped exclusive breastfeeding at 6 month or later	-	129/529 (24.4)	215/692 (31.1)	142/397 (35.8)	-	289/1435 (20.1)	256/1051 (24.4)	98/374 (26.2)	-	418/1964 (21.3)	471/1743 (27.0)	240/771 (31.1)
missing	0/433 (0.0)	1/529 (0.2)	3/692 (0.4)	2/397 (0.5)	1/1511 (0.1)	0/1435 (0.0)	3/1051 (0.3)	1/374 (0.3)	1/1944 (0.1)	1/1964 (0.1)	10/1743 (0.6)	3/771 (0.4)
Height/length for age (stunting) (z-score)	-0.17 (-1.10,0.49)	-0.99 (-1.77,-0.16)	-1.46 (-2.15,-0.83)	-1.21 (-1.73,-0.56)	-0.46 (-1.32,0.39)	-0.98 (-1.79,-0.16)	-1.20 (-2.13,-0.32)	-0.86 (-1.58,-0.13)	-0.40 (-1.25,0.43)	-0.99 (-1.77,-0.16)	-1.34 (-2.13,-0.53)	-1.11 (-1.67,-0.33)
Stunted N (%)	34/433 (7.9)	86/529 (16.3)	193/692 (27.9)	63/397 (15.9)	168/1503 (11.2)	259/1435 (18.1)	284/1051 (27.0)	59/374 (15.8)	202/1936 (10.4)	345/1964 (17.6)	477/1743 (27.4)	122/771
Missing	5/433 (1.2)	7/529 (1.3)	18/692 (2.6)	8/397 (2.0)	22/1511 (1.5)	34/1453 (2.3)	39/1076 (3.6)	24/391 (6.1)	27/1944 (1.4)	41/1982 (2.1)	57/1768 (3.2)	32/788 (4.1)
Weight for height/length (wasting) (z-score)	-0.77 (-1.58,0.01)	-1.0 (-1.81,-0.21)	-0.72 (-1.42,0.02)	-0.80 (-1.38,-0.23)	0.10 (-0.72,0.89)	-0.21 (-1.03,0.58)	-0.42 (-1.17,0.33)	-0.65 (-1.35,0.10)	-0.10 (-0.94,0.75)	-0.43 (-1.27,0.37)	-0.54 (-1.30,0.22)	-0.73 (-1.35,-0.09)
Wasted N (%)	68/433 (15.7)	108/529 (20.4)	76/692 (11.0)	44/397 (11.1)	74/1503 (4.9)	102/1435 (7.1)	87/1051 (8.3)	30/374 (8.0)	142/1936 (7.3)	210/1964 (10.7)	163/1743 (9.4)	74/771 (9.6)
Missing	7/433 (1.6)	7/529 (1.3)	18/692 (2.6)	7/397 (1.8)	23/1511 (1.5)	34/1453 (2.3)	39/1076 (3.6)	24/391 (6.1)	30/1944 (1.5)	41/1982 (2.1)	57/1768 (3.2)	31/788 (3.9)
**MUAC z score**	-0.38 (-1.04,0.25)	-0.68 (-1.30,0.03)	-0.98 (-1.58,-0.36)	-0.84 (-1.38,-0.29)	0.24 (-0.51,0.98)	-0.10 (-0.83,0.72)	-0.40 (-1.05,0.28)	-0.47 (-1.11,0.34)	0.05 (-0.64,0.83)	-0.24 (-1.0,0.56)	-0.61 (-1.31,0.08)	-0.64 (-1.26,-0.04)
MUAC under threshold	27/433 (6.2)	49/529 (9.3)	93/692 (13.4)	44/397 (11.1)	47/1503 (3.1)	73/1435 (5.1)	52/1051 (5.0)	24/374 (6.4)	74/1936 (3.8)	122/1964 (6.2)	145/1743 (8.3)	68/771 (8.8)
<11.5cm (severe malnutrition)	23/433 (5.3)	16/529 (3.0)	10/692 (1.4)	2/397 (0.5)	62/1503 (4.1)	19/1435 (1.3)	9/1051 (0.9)	3/374 (0.8)	85/1936 (4.4)	35/1964 (1.8)	19/1743 (1.1)	5/771 (0.6)

Between 11.5 and 12.5cm (moderate malnutrition)	62/433 (14.3)	60/529 (11.3)	47/692 (6.8)	7/397 (1.8)	176/1503 (11.7)	80/1435 (5.6)	22/1051 (2.1)	2/374 (0.5)	238/1936 (12.3)	140/1964 (7.1)	69/1743 (4.0)	9/771 (1.2)
Missing	0/433 (0.0)	2/529 (0.4)	6/692 (0.9)	7/397 (1.8)	10/1511 (0.7)	21/1453 (1.4)	29/1076 (2.7)	19/391 (4.9)	10/1944 (0.5)	23/1982 (1.2)	35/1768 (2.0)	26/788 (3.3)
Neuro Assessment N (%)												
number of children assessed with MDAT	427	523	678	390	1493	1418	1015	369	1920	1941	1693	759
Screened positive MDAT <-1SD	88 (20.6)	48 (9.2)	85 (12.5)	55 (14.1)	147 (9.8)	128 (9.0)	105 (10.3)	29 (7.9)	235 (12.2)	176 (9.1)	190 (11.2)	84 (11.1)
Screened positive MDAT <-2SD	16 (3.7)	11 (2.1)	12 (1.8)	8 (2.1)	30 (2.0)	41 (2.9)	26 (2.6)	16 (4.3)	46 (2.4)	52 (2.7)	38 (2.2)	24 (3.2)
number of children assessed with OMCI	428	-	678	-	1498	-	1018	-	1917	-	1696	-
number of children assessed with GMA video	433	-	-	-	1501	-	-	-	1934	-	-	-
number of children assessed with Family Care Indicators questionnaire	-	528	690	395	-	1435	1051	374	-	1963	1741	769
number of children assessed with NDST	-	-	689	394	-	-	1050	374	-	-	1739	768
At risk of developmental delay	-	-	15/689 (2.2)	3/395 (0.8)	-	-	57/1051 (5.4)	25/374 (6.7)	-	-	72/1740 (4.1)	28/769 (3.6)
Screened positive NDST												
number of children assessed with epilepsy questionnaire	-	-	689	394	-	-	1051	374	-	-	1739	768
Screened positive epilepsy	-	-	3 (0.4)	1 (0.3)	-	-	35/1051 (3.3)	16/374 (4.3)	-	-	38/1739 (2.2)	17/768 (2.2)
Number of children flagged during the study*			16/689 (2.3)	4/394 (1.0)			59/1050 (5.6)	25/369 (6.8)			75/1739 (4.3)	29/768 (3.8)
number of children assessed with PedSQL	-	-	16/16	4/4	-	-	59/59	25/25	-	-	75/75	29/29
number of children Screened positive for MCHAT	-	-	9/16 (56.3)	1/4 (25.0)	-	-	14/59 (23.7)	3/25 (12.0)	-	-	23/75 (30.7)	4/29 (13.8)
number of children Screened positive for CARDIF		-	15/16 (93.8)	2/4 (50.0)	-	-	52/59 (88.1)	23/25 (92.0)	-	-	67/75 (89.3)	25/29 (86.2)
**Number of phone interview**	-	-	-	-	8/1511 (0.5)	18/1453 (1.2)	26/1076 (2.4)	17/391 (4.3)	8/1944 (0.4)	18/1982 (0.9)	26/1768 (1.5)	17/788 (2.2)
number of children assessed with Developmental Milestones Checklist (DMC-III)	-	0 (0.0)	-	-	8/8 (100.0)	18/18 (100.0)	26/26 (100.0)	17/17 (100.0)	8/8 (100.0)	18/18 (100.0)	26/26 (100.0)	17/17 (100.0)

***Children flagged during the study for assessment with MDAT and NDST were asked the following questionnaire: PedSQL, MCHAT, and CARDIF**

Table S4 Pregnancy outcomes of participants recruited **to quality of care and health economics sub-studies**

Quality of care	The Gambia	Kenya	All countries
**Number of participants**	437	695	1132
**Controls: Uncomplicated pregnancy** N (%)	210 (48.0)	328 (47.2)	538 (47.5)
**Cases** N (%)			
Stage 2 Hypertension	16 (3.7)	47 (6.8)	63 (5.6)
Caesarean section	21 (4.8)	117 (16.8)	138 (12.2)
Stillbirth	19 (4.3)	12 (1.7)	31 (2.7)
Neonatal death	1 (0.2)	4 (0.6)	5 (0.4)
Small vulnerable newborn	194 (44.4)	279 (40.1)	473 (41.8)
Small for gestational age <3rd percentile	39 (8.9)	57 (8.2)	96 (8.5)
Preterm birth <33 weeks	20 (4.6)	33 (4.7)	53 (4.7)
**Health Economics**	The Gambia	Kenya	All countries
**Number of participants**	110	100	210
**Controls: Uncomplicated pregnancy** N (%)	56 (50.9)	46 (46.0)	102 (48.6)
**Cases** N (%)			
Stage 2 hypertension	2 (1.8)	3 (3.0)	5 (2.4)
Caesarean section	5 (4.5)	12 (12.0)	17 (8.1)
Stillbirth	7 (6.4)	0 (0.0)	7 (3.3)
Neonatal death	0 (0.0)	3 (3.0)	3 (1.4)
Small vulnerable newborn	35 (31.8)	39 (39.0)	74 (35.2)
Small for gestational age <3rd percentile	13 (11.8)	5 (5.0)	18 (8.6)
Preterm birth <33 weeks	3 (2.7)	4 (4.0)	7 (3.3)

Table S5 Number of aliquots collected for each sample type by study visit

	Gambia				Kenya				Total			
**Women**	**Visit 1 (6 wk-6 months after birth)**	**Visit 2 (12 months after birth)**	**Visit 3 (24 months after birth)**	**Visit 4 (36 months after birth)**	**Visit 1 (6 wk-6 months after birth)**	**Visit 2 (12 months after birth)**	**Visit 3 (24 months after birth)**	**Visit 4 (36 months after birth)**	**Visit 1 (6 wk-6 months after birth)**	**Visit 2 (12 months after birth)**	**Visit 3 (24 months after birth)**	**Visit 4 (36 months after birth)**
Whole blood	426	520	660	383	985	1355	935	319	1411	1875	1595	702
Blood spot		519				1161				1680		
Serum	2033	2347	2956	1750	5325	7321	5041	1700	7358	9668	7997	3450
Plasma	2260	2555	3239	1883	5615	7768	5422	1826	7875	10323	8661	3709
Buffy coat	426	520	659	383	985	1355	934	319	1411	1875	1593	702
Urine	772	1072	1363	0	2020	2792	9160	0	2792	3864	10523	0
Vaginal swab - biochemistry	1507								1507			
Vaginal swab - microbiome	542								542			
Breastmilk (Gambia only)	1986								1986			
**Children**	**Visit 1**	**Visit 2**	**Visit 3**	**Visit 4**	**Visit 1**	**Visit 2**	**Visit 3**	**Visit 4**	**Visit 1**	**Visit 2**	**Visit 3**	**Visit 4**
Blood spot	429	519	507	188	1290	373	188	47	1719	892	695	235
Serum		2	263	323		1472	1184	469		1474	1447	792
Plasma		2	350	433		2169	1765	706		2171	2115	1139
Buffy coat		1	170	188		771	628	247		772	798	435
Stool	190		82		646		196		836		278	
