# Supplementary material for: PRECISE-DYAD: a prospective cohort study linking maternal and infant health trajectories in sub-Saharan Africa
Source: BMJ Open. 2026 Jul 17;16(7):e115586. doi: 10.1136/bmjopen-2025-115586 (PMC13384177; doi:10.1136/bmjopen-2025-115586)
Supplement: Supplementary file 6 [file bmjopen-2025-115586.reviewer_comments.pdf]

PEER REVIEW HISTORY

BMJ Open publishes all reviews undertaken for accepted manuscripts. Reviewers are asked to complete a checklist review form and are provided with free text boxes to elaborate on their assessment. These free text comments are reproduced below.

ARTICLE DETAILS

Title (Provisional)

Cohort Profile: PRECISE-DYAD: a prospective cohort study linking maternal and infant health trajectories in sub-Saharan Africa

Authors

Volvert, Marie-Laure; Wilson, Milly; Owino, Robin Okello; Blencowe, Hannah; Koech, Angela; Jah, Hawanatu; Idris, Yahaya; Wanje , Onesmus; Mwaniki, Isaac Gathogo; Mutunga, Joseph; Touray, Fatima; Mwadime , Emily; Roca, Anna; Omuse, Geoffrey; Craik, Rachel; Kongira, Fatoumata; Mukhanya, Moses; Bojang , Kalilu; Bah, Baboucar; Ochieng , Marvin; D'Alessandro, Umberto; Mwashigadi, Grace; Mutua , Agnes Mutindi; Rerimoi , Anne; Temmerman, Marleen; Akuze, Joseph; Martinez-Alvarez, M; Magai, Dorcas N; Barratt, Ben; Bah, Yorro; Li, Jing; Chandna, Jaya; Gladstone, Melissa; Abubakar, Amina; Tribe, Rachel M.; Khalil, Asma; Makanga, Prestige Tatenda; Salisbury, Tatiana; Mistry, Hiten; Moore, Sophie; Nabwera, Helen; Filippi, Veronique; Magee, Laura A.; Vidler, Marianne; Makacha, Liberty; Poston, Lucilla; Sevene, Esperanca; von Dadelszen, Peter; and the PRECISE Network , ,

---

VERSION 1 - REVIEW

**Reviewer**	**1**
**Name**	**Helou, Amyna**
**Affiliation**	**Monash University, Centre for Medicine Use and Safety**
**Date**	**02-Apr-2026**
**COI**	**None**

Thank you for the opportunity to review this important manuscript. Overall, the manuscript is well written. Please consider the following minor revisions to refine this manuscript:

On Page 14 of 51 line 34-35 the following is mentioned:

" (in this manuscript we have excluded the Mozambican data)"

However, on page 16 of 51 line 35 under the air quality sub-study, Mozambique is mentioned as one of the locations the 343 women were recruited from. The data is also presented in Table 5. Please clarify this point on page 14 as mentioned above, or remove the Mozambican data as air quality is the only reported data point in this manuscript.

- page 16 of 51 line 35, 'recruited' is repeated, please remove one

-p16 of 51 line 52 "Second PRECISE Pregnancies", was there going to be more writing after this?

Page 18 of 51 line 48 "[37]" is typed twice, please remove one

---

**Reviewer**	**2**
**Name**	**Kumma, Wondimagegn**
**Affiliation**	**Wolaita Sodo University, Public Health**
**Date**	**13-Apr-2026**
**COI**	**None**

Review of manuscript with ID bmjopen-2025-115586

This is a massive study with a cohort design. It investigates multiple outcome variables and is advantageous to address several maternal and child health-related problems. Therefore, I encourage proceeding with publication with major corrections.

Descriptions of data collection are shallow and require readers to refer to different documents published prior to this paper, as the numbers of variables for measurement are many and require clarity. In addition, there is a need for extensive investigation for overlapping reporting findings. Errors related to typographical issues, such as repeated words as indicated on line number 35, need to be corrected.

I recommend providing more detailed information on the study setting, as the study is a cohort and multi-center. The description of the study population is clear, but there are sizable dropout rates or loss to follow-up at different stages of follow-up visits. The ethical consideration requires extensive description, as the research involves human subjects with sensitive data collection, such as biological data. The section meant for ethical consideration should clearly indicate consent and assent, voluntary involvement, the right to withdraw from the study, and anonymity. In this study, there are two main outcome variables that require a clear definition. The underdescription of the outcome variables creates a lack of clarity regarding the measurement. The readers do not need to refer to another paper while reading this paper. The statistics lacked a dedicated section and were presented unclearly in the paper. The paper indicates that the study is descriptive, but it should clearly depict the descriptive analysis. The discussion did not clearly consist of the important findings of the study. They focused more on the limitation than the result of the study. The discussion did not focus on the main findings of the study in detail.

VERSION 1 - AUTHOR RESPONSE

Reviewer: 1
Dr. Amyna Helou, Monash University
Comments to the Author:
Thank you for the opportunity to review this important manuscript. Overall, the manuscript is well written. Please consider the following minor revisions to refine this manuscript:

On Page 14 of 51 line 34-35 the following is mentioned:
" (in this manuscript we have excluded the Mozambican data)"
However, on page 16 of 51 line 35 under the air quality sub-study, Mozambique is mentioned as one of the locations the 343 women were recruited from. The data is also presented in Table 5. Please clarify this point on page 14 as mentioned above, or remove the Mozambican data as air quality is the only reported data point in this manuscript.

Response: Thank you very much for carefully reading our manuscript and for your helpful feedback. The PRECISE study was conducted in three countries: Kenya, The Gambia, and Mozambique. The PRECISE-DYAD study was a follow-up study conducted only in Kenya and The Gambia, and did not include Mozambique.

We agree that the sentence "in this manuscript we have excluded the Mozambican data" was potentially confusing and have therefore removed it. We have replaced it with "PRECISE cohorts from Kenya and The Gambia" to clarify that only these two cohorts are represented in this table. We hope this revision improves clarity.

We would like to retain the Mozambican data in the Air quality as this was previously described in the Protocol (Craik et al., Wellcome Open Research, 2021).

- page 16 of 51 line 35, 'recruited' is repeated, please remove one

-p16 of 51 line 52 "Second PRECISE Pregnancies", was there going to be more writing after this?

Page 18 of 51 line 48 "[37]" is typed twice, please remove one

Thank you for identifying these errors. The manuscript has been corrected accordingly.

Reviewer: 2
Dr. Wondimagegn Kumma, Wolaita Sodo University
Comments to the Author:
Review of manuscript with ID bmjopen-2025-115586
This is a massive study with a cohort design. It investigates multiple outcome variables and is advantageous to address several maternal and child health-related problems. Therefore, I encourage proceeding with publication with major corrections.

Descriptions of data collection are shallow and require readers to refer to different documents published prior to this paper, as the numbers of variables for measurement are many and require clarity. In addition, there is a need for extensive investigation for overlapping reporting findings.

Thank you for reading our manuscript and providing this helpful feedback. We have added Table 1a and 1b describing the data and sample collection performed at each visit. We hope these additions

improve clarity for the reader. These tables are also available in our study protocol (Craik et al., Wellcome Open Research, 2021)

Errors related to typographical issues, such as repeated words as indicated on line number 35, need to be corrected.

Thank you for identifying this error, it has now been corrected.

I recommend providing more detailed information on the study setting, as the study is a cohort and multi-center. The description of the study population is clear, but there are sizable dropout rates or loss to follow-up at different stages of follow-up visits.

Thank you for your suggestion. We have now added maps illustrating the study settings. We have described the study settings consistently with the previously published PRECISE protocol (von Dadelszen et al., Reproductive Health, 2020, 17(Suppl 1):51) and PRECISE-DYAD protocol (Craik et al., Wellcome Open Research, 2021).

Regarding the loss to follow-up across study visits, we acknowledge that attrition occurred at different stages of follow-up, which is common in longitudinal cohort studies conducted in low-resource settings. We described participant follow-up and retention across visits within the manuscript for transparency for readers.

The ethical consideration requires extensive description, as the research involves human subjects with sensitive data collection, such as biological data. The section meant for ethical consideration should clearly indicate consent and assent, voluntary involvement, the right to withdraw from the study, and anonymity.

Thank you, we have added a line regarding consent in the ethical section. We have previously published a full protocol paper including more details of the consent process, (Craik et al., Wellcome Open Research, 2021).

In this study, there are two main outcome variables that require a clear definition. The under description of the outcome variables creates a lack of clarity regarding the measurement. The readers do not need to refer to another paper while reading this paper. The statistics lacked a dedicated section and were presented unclearly in the paper. The paper indicates that the study is descriptive, but it should clearly depict the descriptive analysis. The discussion did not clearly consist of the important findings of the study. They focused more on the limitation than the result of the study. The discussion did not focus on the main findings of the study in detail.
